# Supplementary material for: High-efficiency broadband achromatic metalens for near-IR biological imaging window
Source: Nat Commun. 2021 Sep 21;12:5560. doi: 10.1038/s41467-021-25797-9 (PMC8455568; doi:10.1038/s41467-021-25797-9)
Supplement: Supplementary file 1 — Supplementary Information [file 41467_2021_25797_MOESM1_ESM.docx]

**Supplementary** **Information for:**

**High-efficiency broadband achromatic metalens for near-IR biological imaging window**

Yujie Wang^1,†^, Qinmiao Chen^1,†^, Wenhong Yang^1,†^, Ziheng Ji^1,†^, Limin Jin^1^, Xing Ma^1^, Qinghai Song^1^, Alexandra Boltasseva^2^, Jiecai Han^3^, Vladimir Shalaev^2^, Shumin Xiao,^1,3,4,*^

1. State Key Laboratory on Tunable laser Technology, Ministry of Industry and Information Technology Key Lab of Micro-Nano Optoelectronic Information System, Shenzhen Graduate School, Harbin Institute of Technology, Shenzhen, 518055, P. R. China.
2. School of Electrical and Computer Engineering and Birck Nanotechnology Center, Purdue University, West Lafayette, 47907 Indiana, USA
3. National Key Laboratory of Science and Technology on Advanced Composites in Special Environments, Harbin Institute of Technology, Harbin 150080, P. R. China.
4. Collaborative Innovation Center of Extreme Optics, Shanxi University, Taiyuan, 030006, Shanxi, P. R. China.

†These authors contributed equally: Yujie Wang, Qinmiao Chen, Wenhong Yang, Ziheng Ji

Corresponding authors: * [shumin.xiao@hit.edu.cn](mailto:shumin.xiao@hit.edu.cn)

Supplementary Text

In the main text, we have reported the high efficiency, polarization insensitive, and broadband achromatic metalens. For the first time, we show that the high quality achromatic TiO_2_ metalens can be comparable to the commercial micro-lenses. In this supplementary information, we show the details of experimental methods, results, and the corresponding numerical calculations.

**Supplementary Note 1: The statistics of focus efficiency of broadband achromatic metalenses**

Achromatic metalens has been intensively studied in the past few years. Despite of a few bi- or tri-wavelength metalens, most of them have not very high focusing efficiencies, far below their commercial counterparts of conventional lens or multi-level diffractive lens. On one hand, this is caused by the un-optimized optical design. On the other hand, it is restricted by the material absorption or the limited group delay range. Fig. 1 of the main text summarizes the focus efficiencies of broadband achromatic metalens reported in literatures. It is easy to see that the focus efficiency of Si metalens works very well for a wide spectral range from 1200 – 1600 nm. The average efficiency is as high as 50% (see squares in Fig. 1 of the main text). However, the applications of Si metalens at shorter wavelength are restricted because the focus efficiency is reduced significantly by the material loss.

TiO_2_ is a kind of high refractive index material that is transparent in visible spectrum and near IR. It has also been widely utilized to construct all-dielectric metalenses. However, this kind of metalens are limited by the nanofabrication technique. The state of art technique only produces TiO_2_ nanopillars with height of 990 nm. Otherwise, random voids will be generated within the nanopillars, destroying the designed group delay and the broadband achromatism. As a result, the experimental efficiency of achromatic TiO_2_ metalens is ~ 70% at 450 nm and reduces to below 30% at red light (dots in Fig. 1 of the main text). Similar situation holds true for the GaN metalenses. Therefore, it is obviously to see a low efficiency gap from 650 nm to 1000 nm, which is also known as the first biological window for optical imaging.

The crosses in Fig. 1 of the main text represent the experimentally measured focus efficiency of the TiO_2_ metalenses reported in the main text. With the increase of pillar height, the average efficiency for metalenses with NA = 0.1 and NA = 0.24 are 88.1% and 77%, respectively. It is important to emphasize two key points of the new TiO_2_ achromatic metalens. 1. It can perfectly cover the low-efficiency gap for the near-IR biological window. The metalens is also independent to the polarization. Thus, the new metalens shall be important for biological applications. 2. The exact efficiencies are much higher than the previous reports. Actually, they are even superior to the commercial micro-lenses. From the point view of applications, this broadband TiO_2_ metalenses can be a game changer on the research field of flat optoelectronics.

**Supplementary Note 2: The libraries of phase and group delay provided by the TiO_2_ nanopillars**

In the main text, the achromatic metalenses are designed following the equation 1 and its Taylor expansion. Basically, to eliminate the achromatic aberration, both of the required phase and the required group delay must be fulfilled. We utilize the transmission phase along the TiO_2_ nanopillars following the equation

$\varphi\left( r,\omega\right)=\frac{\omega}{c}n_{eff}H$*,*  (1)

where *H* represent height of TiO_2_ structure, and *n_eff_* represent the effective index. The unit placed at a radial coordinate *r* provides the same phase so that different wavelengths are deflected by the same angle. In order to achieve achromatic focusing, the unit needs to satisfy not only the required phase, but also the group delay. The group delay is the derivative with respect to angular frequency:

$\frac{\partial\varphi\left( r,\omega\right)}{\partial\omega}=\frac{1}{c}n_{eff}H+\frac{\omega}{c}\frac{\partial n_{eff}}{\partial\omega}H$ (2)

The group delay can thus be expanded by increasing the height of nanopillar and the range of effective index. When the pillar height is fixed, the group delay is sensitive to the effective index, which is dependent on the geometry and dimensions in period.


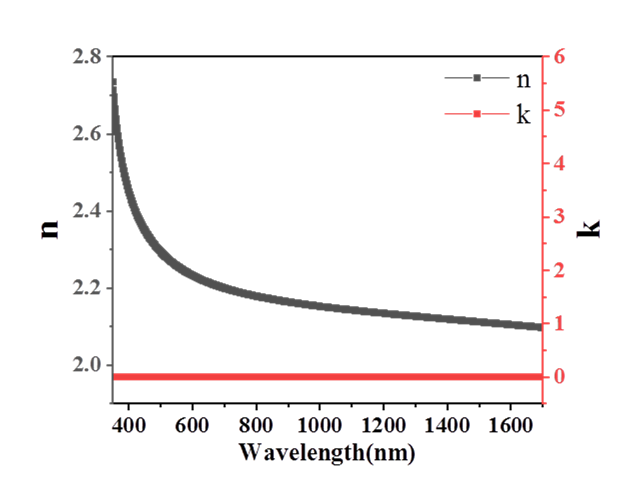


**Supplementary Figure 1. The refractive index and light extinction coefficient of TiO_2_ film.**

Since we are aiming at the biological imaging window, several key issues must be considered, e.g., the material absorption, the polarization dependence, the efficiency and the achromatic spectral range. Consequently, TiO_2_ nanopillars with at least 4-fold rotational symmetry are selected to eliminate the polarization dependence, the material absorption, and to effectively collect the signals. We have increased the pillar height to 1500 nm and fixed the cross sections of nanopillars to four fundamental types, i.e. circle, ring, square and bipolar concentric ring. The phase shift and the group delay of nanopillars with different in-plane sizes are calculated with the finite element methods. The refractive index of TiO_2_ film was taken from the optical measurement with the ellipsometer. As shown in Supplementary Figure 1, the TiO_2_ has relatively large refractive index of n > 2.17 for a large spectral range. The imaginary part of refractive index is negligible in the visible and near infrared spectrum. Supplementary Figure 2a shows the numerical results. The group delay We can see that the phase shift covers 0 - 2π. Meanwhile, the group delay varies between 0 fs and 7 fs. Such kind of library is good enough for generating a broadband, polarization insensitive, large NA and high efficiency metalens.

The efficiency is another important parameter for the design of achromatic metalens. When the pillar height is small such as 600 -800 nm, there is only small range of parameters that can generate the required group delays of 5-7 fs. These parameters typically correspond to the resonant dips or low polarization conversion efficiencies. Once such nanopillars are selected, the overall efficiency of the achromatic metalens reduces quickly. When the pillar height is increased to 1500 nm, the parameter space can be dramatically increased. Supplementary Figure 2b shows the transmittance of 2445 TiO_2_ nanopillars as a function of the group delay. While more nanopillars with lower efficiencies appears at larger group delay, there are still numerous TiO_2_ nanopillars that can support high efficiency and the required group delay simultaneously. As a result, the overall efficiency of the metalens can be maintained at a high value for the entire spectral range of the first biological window for optical imaging.


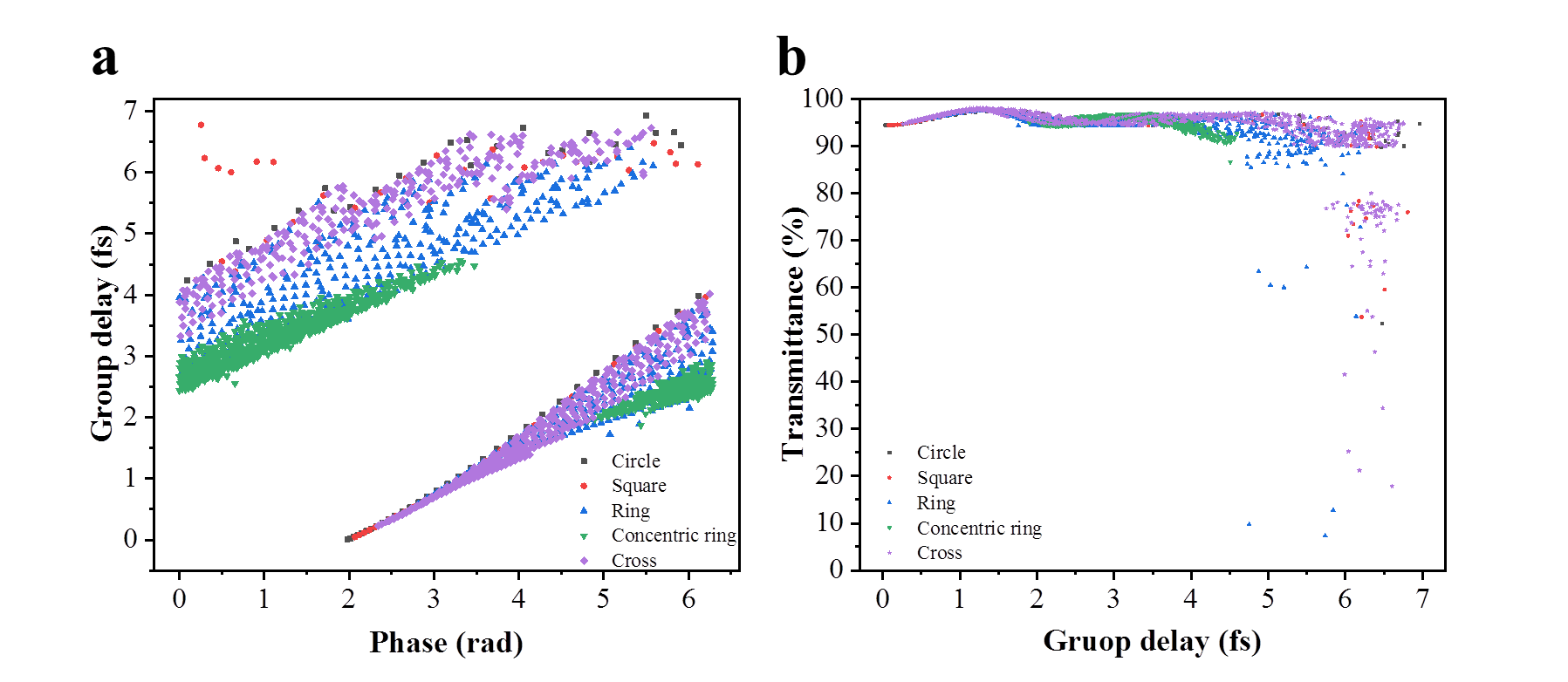


**Supplementary Figure 2. a** The phase shift and group delay libraries. **b** The group delay and transmittance libraries. The contributions of different unit cells are labelled with different colors.

**Supplementary Note 3: The achromatic metalens with NA = 0.1**

In the main text, we have shown two sets of experimental data. One is for the TiO_2_ metalens with NA = 0.24. The other one is for metalens with NA = 0.1. Most of the results for large NA metalens are shown in the main text. Here we show the details of the small NA one. Supplementary Figure 3a shows a quarter of the layout of the metalens. The diameter of the metalens is 25 µm and the thickness of sample is 1500 nm. Since NA is much smaller, three types of fundamental building blocks are good enough to match the required phase shift and group delay. The solid line in Supplementary Figure 3b is the required group delay calculated according to Eq. (1) in the main text and its Taylor expansion. The squares in Supplementary Figure 3b are the selected values that can be provided by the TiO_2_ nanopillars in libraries of Supplementary Figure 2. As the diameter and the NA are reduced, the required group delay is also decreased. As shown in Supplementary Figure 3, this metalens can be fitted much better than the one in the main text. As a result, the average focus efficiency of achromatic metalens has been improved to ~ 93% in the main text. This is also consistent with the previous reports where metalens with smaller NA always have higher efficiency.


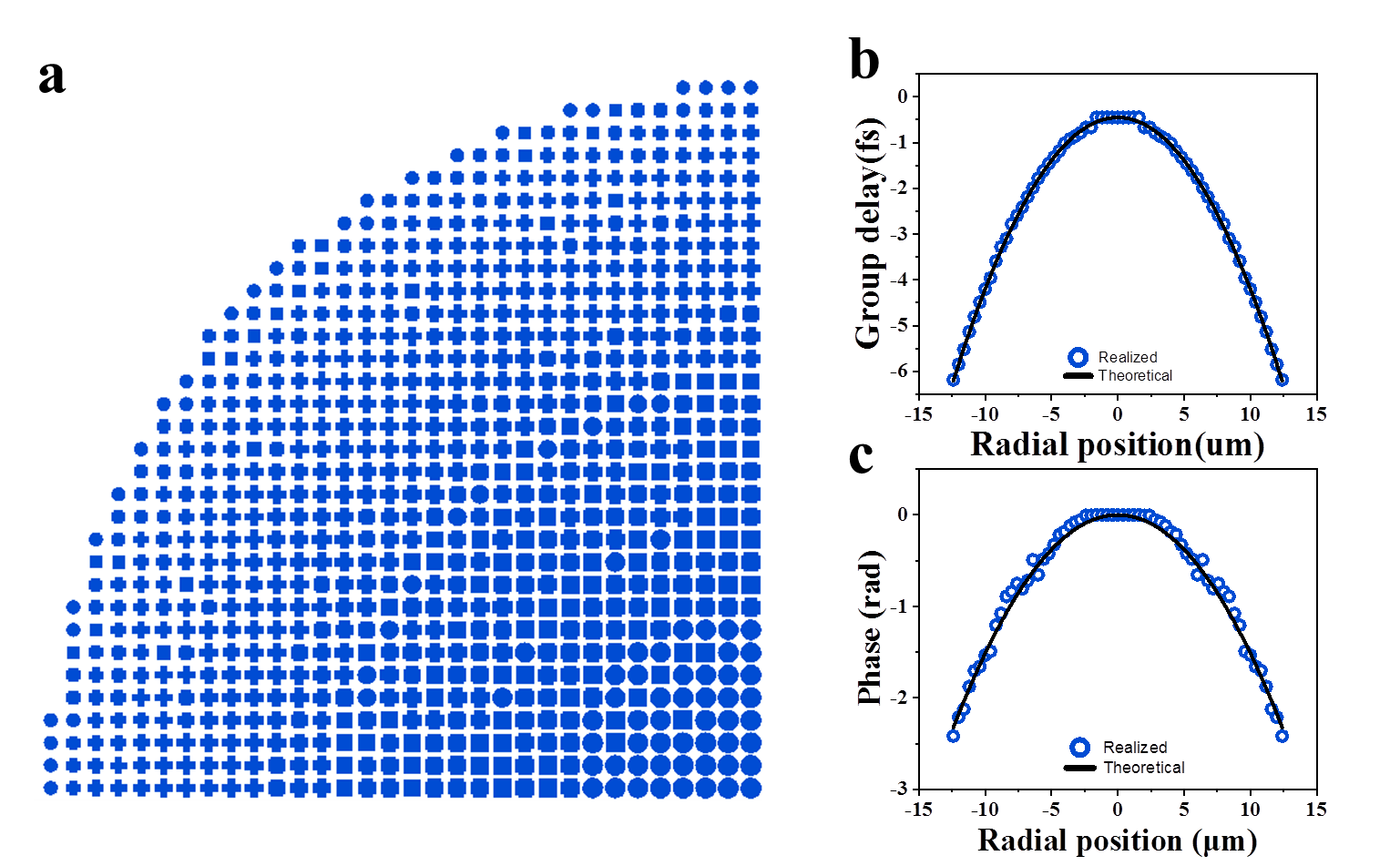


**Supplementary Figure 3. The design of TiO_2_ metalens with NA = 0.1. a** The layout of a quarter of the metalens. Three types of fundamental building blocks are employed, i.e. circle, square, and cross. **b** The required group delay (solid line) and the provided values (dots) from the TiO_2_ nanopillars. **c** The required phase shift (solid line) and the provided values (dots) from the TiO2 nanopillars

Then the TiO_2_ metalens with NA = 0.1 has also been fabricated with the same process described in the methods. In additional to the efficiency shown in the main text, here we show some other related information. Supplementary Figure 4a is the top-view scanning electron microscope (SEM) image of the sample. The enlarged SEM image (Supplementary Figure 4b) with higher resolution clearly show the four fundamental building blocks in the design. The tilt-view SEM image in Supplementary Figure 4c further shows the sidewalls are nearly perfect in the vertical direction. The measured tilt angle is about 89^o^ – 90^o^. Once again, the high quality of our nanofabrication process has been experimentally confirmed. They are also consistent with the record high efficiency in the main text as well.


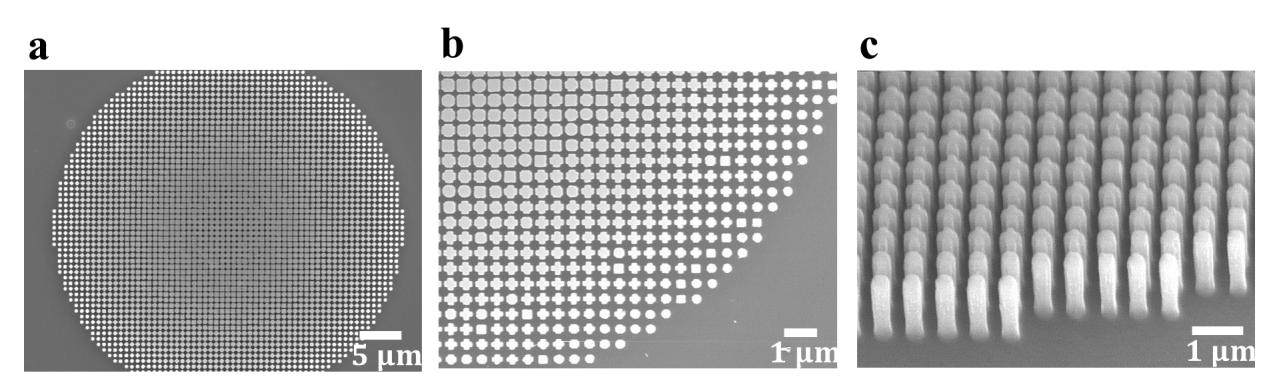


**Supplementary Figure 4. The SEM images of TiO_2_ metalens. a** The top-view SEM image of the entire metalens. **b** The high-resolution top-view SEM image of part of metalens. **c** The tilt-view SEM image of the metalens.

**Supplementary Note 4: The fabrication process of the TiO_2_ metalens**

In the main text and the method section, we have described the fabrication process of the TiO_2_ metalenses. Here we show the detailed process in Fig. 2A of the main text, which can be separated into 8 steps. (1) The 13 nm ITO-coated glass substrate is cleaned in acetone, methanol, and isopropanol. (2) 1500 nm TiO_2_ film was deposited onto the substrate with electron beam evaporation. (3) 200 nm PMMA A2 (Microchem) electron-beam resist is spin-coated onto the TiO_2_ membrane. (4) The PMMA resist is patterned in electron beam aligner. The inversed pattern is generated by developing in MIBK/IPA for 30 seconds. (5) 30 nm Cr is highly directionally deposited onto the nanopattern via electron-beam evaporation. (6) The PMMA resist is removed and the nanopattern is transferred into the Cr. (7) Taking Cr as hard mask, the TiO_2_ is etched with reactive ion etching process. (8) After removing the Cr mask, the TiO_2_ metalens is finally achieved.

The RIE process for TiO_2_ has been studied for many years. However, the previous recipe only produces a sidewall with tilt angle of < 75^o^, strongly restricting the applications in high quality flat devices such as metalenses.

Supplementary Figure 4 shows the tilt view SEM images of the TiO_2_ nanostructures. In Supplementary Figure 5a, there are many types of nanostructures including circular nanopillars, nanostripes, rectangle nanopillars, as well as nano-crosses. While the geometries and the pillar sizes are all different, we can see that the nanostructures are well kept and their sidewalls follow 90^o^ in vertical direction very well. Supplementary Figure 5b shows the high-resolution SEM image of the meta-crosses. The width of each arm is 40 nm. Comparing with the pillar height (1500 nm), the aspect ratio can be as high as 37.5. The nanopillar in Supplementary Figure 5c has rectangle cross sections. Similar high quality has also been held. Consequently, we can conclude that our new technology is suitable for high aspect ratio and large height TiO_2_ nanostructures. This is an important basis for the research on high efficiency achromatic metalens. Meanwhile, the results in Supplementary Figure 5c shows that it is also applicable to Pancharatnam−Berry (PB) phase metasurfaces.


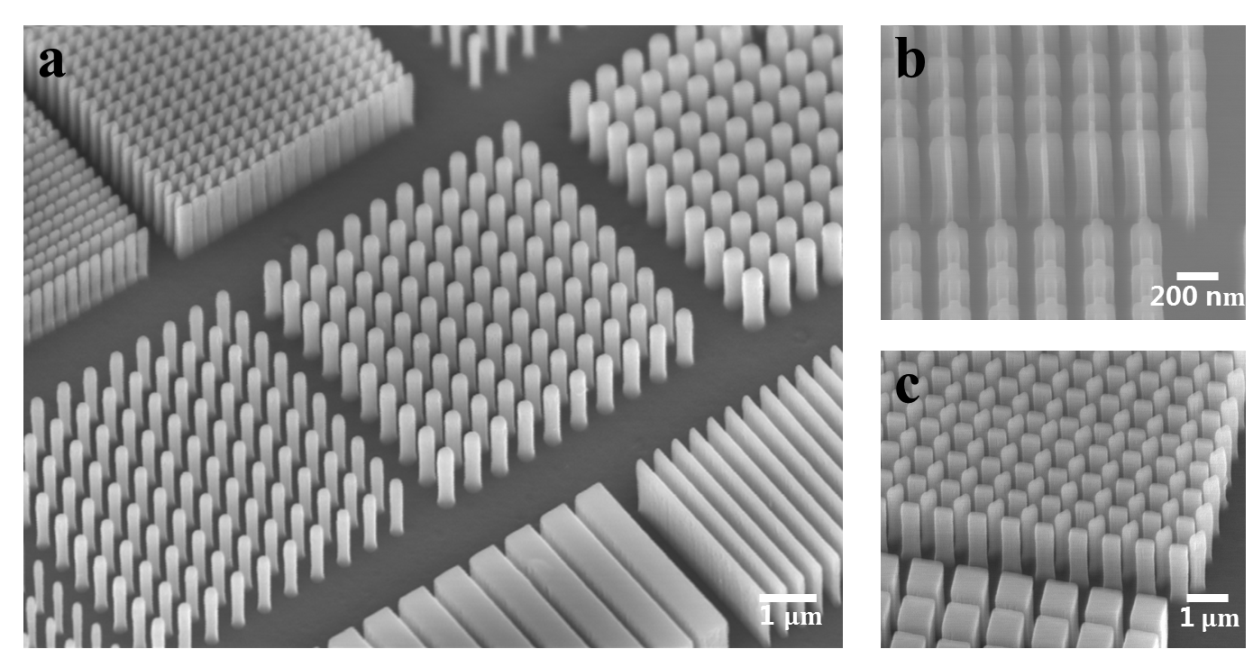


**Supplementary Figure 5. The tilt view SEM images of the TiO_2_ nanostructures. a** The SEM of different nanostructures including circular pillars, crosses, as well as rectangle pillars. **b** and **c** are the high-resolution SEM images of the crosses and the rectangle pillars.

**Supplementary Note 5: The optical setups**

Supplementary Figure 6 depicts the schematic of the optical setup that characterizes the TiO_2_ metalens. Basically, a supercontinuum laser is expanded to a collimated beam with a diameter of 5 mm to count the deflection loss at the metalens edge and to avoid the overestimation. After the beam passes the substrate, the light in the metalens region is focused to a diffraction limit spot. In case of narrow band characterization, a bandpass filter can also be applied between metalens and the laser source. The focal point is characterized by a commercial objective lens and coupled to a camera by an additional lens. The profiles of the focal spot can be obtained by moving the objective lens with a three-dimensional translation stage. The light out of the metalens region propagates to the forward direction and won’t be collected by the objective lens.


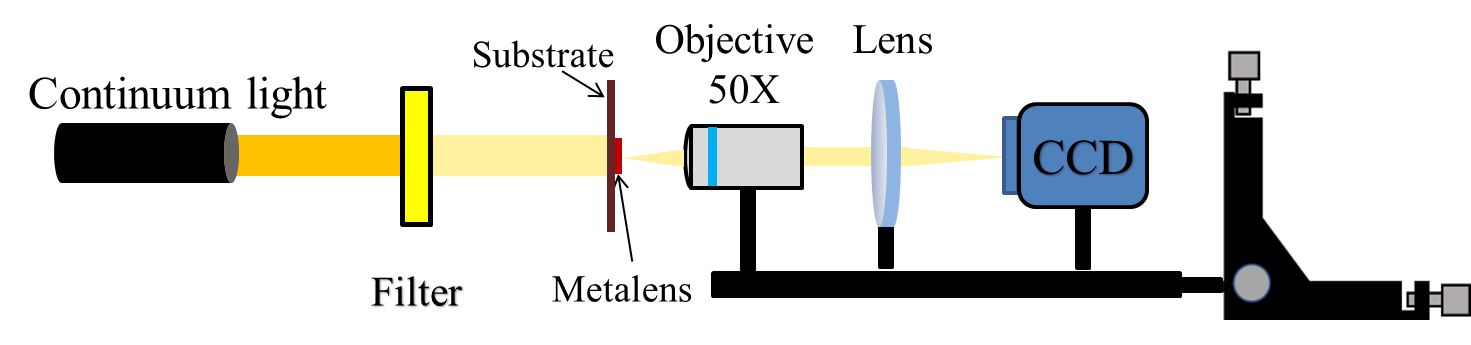


**Supplementary Figure 6.** **The optical setup for characterization of TiO_2_ metalens.** Here metalens is placed at the focal plane of 50X achromatic objective lens. All the optical components are achromatic in the operation wavelength range.

When the focusing efficiency is measured, a CCD camera is applied. The metalens itself and the light field passing through it can be captured by the imaging system including the objective lens, collection lens, and CCD camera. We first image the metalens and mark the covering region. Then the metalens is in-plane shifted 1 mm. In this case, the incident beam can be captured and the overall intensity is achieved by integrating the intensity within the marked region. The metalens is moved back and the tune the camera to the focal plane of metalens. A bright focal spot can be capture by the CCD camera. To accurately capture the intensity of focal spot, we integrate a region slightly larger than the focal point (3 times of its FWHM). In this experiment, the background is carefully subtracted and the intensity is controlled to be below the saturation of CCD camera.

Supplementary Figure 7 shows the optical setup for the optical measurement of the 1951 USAF resolution test chart. It is quite similar to Supplementary Figure 6 except several differences. A supercontinuum laser is focused by a metalens to a diffraction limit spot. In case of narrow band characterization, a bandpass filter can also be applied between metalens and the laser source. The transmitted light through 1951 USAF resolution test chart is collected by a commercial objective lens and imaged to a camera by an additional lens.


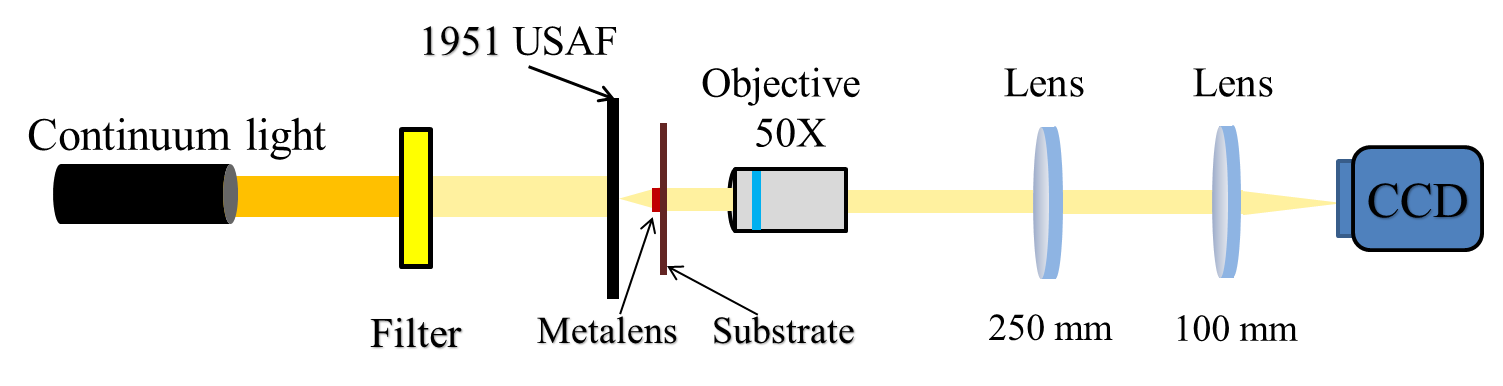


**Supplementary Figure 7.** **The optical setup for measurement of the 1951 USAF resolution test chart using TiO_2_ metalens.** All the optical components are achromatic in the operation wavelength range.

**Supplementary Note 6: The polarization dependence**

In the main text, we have shown that the high efficiency metalenses at 800 nm are independent on the polarization state of incident light. Here we further demonstrate the polarization insensitivity at all the other wavelengths. The results are shown in Supplementary Figure 8. It is easy to see that both X-polarization light and Y-polarization light give the same focus efficiencies at all the wavelengths as the unpolarized incident light. This is a direct proof of the broadband polarization insensitivity.


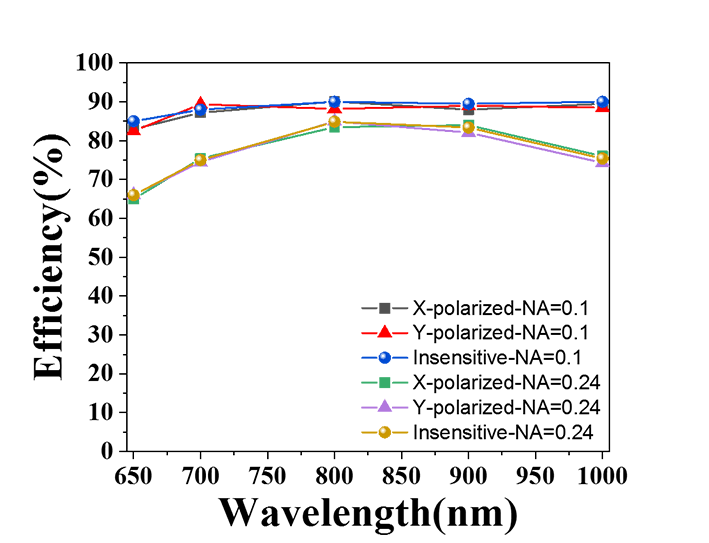


**Supplementary Figure 8.** **The dependence of focus efficiencies of metalenses with NA = 0.1 and 0.24 at all wavelengths on the polarizations of incident light.**

**Supplementary Note 7: The upconversion fluorescent imaging**

In the main text, we have briefly described the measurement of up-conversion imaging with the achromatic metalens. Here we show the experimental details. Supplementary Figure 9a shows the schematic picture of the optical setup for upconversion fluorescent imaging with metalens. The 980 nm laser was spatially filtered into Gaussian profile, then focused to the back focal plane of 50X objective lens by a 300 mm lens, forming a ~50 µm collimated excitation beam at the focal plane of 50X OL to overfill metalens aperture. When the system is working in the monitor module, CCD-2 is placed at 1f position after 250 mm doublet lens, thus conjugated with 50X objective lens focal plane. This module is applied to align metalens and 980 excitation beam, control distance between metalens and sample. Then the system is switched to the confocal module (defined by Metalens, 50X objective lens, 250 mm doublet lens, 100 mm lens and CCD-1) with the flip mirror. Here CCD-1 is placed at 1f position after 100 mm lens, thus conjugated with metalens focal plane. The photoluminescence from the upconversion nanocrystals (NCs) are collected by the same metalens and captured by the CCD-1 camera. A virtual confocal pinhole on the camera will be defined with a sized of 1/3 Airy Unit. Only digital counts within virtual pinhole will be recorded. By scanning the three-dimensional translation state, the two-photon imaging of the NCs based microplate can be monitored even though the microplate is buried by the polystyrene spheres. Supplementary Figure 9b shows the optical setup for the conventional confocal system with objective lens. By removing 300 mm lens in Supplementary Figure 9a, a classical confocal microscope is formed. Here the monitor module can also be saved. Basically, 980 nm laser over filled the 10X achromatic objective lens. The upconversion signals are collected by the objective lens and monitored by the CCD.


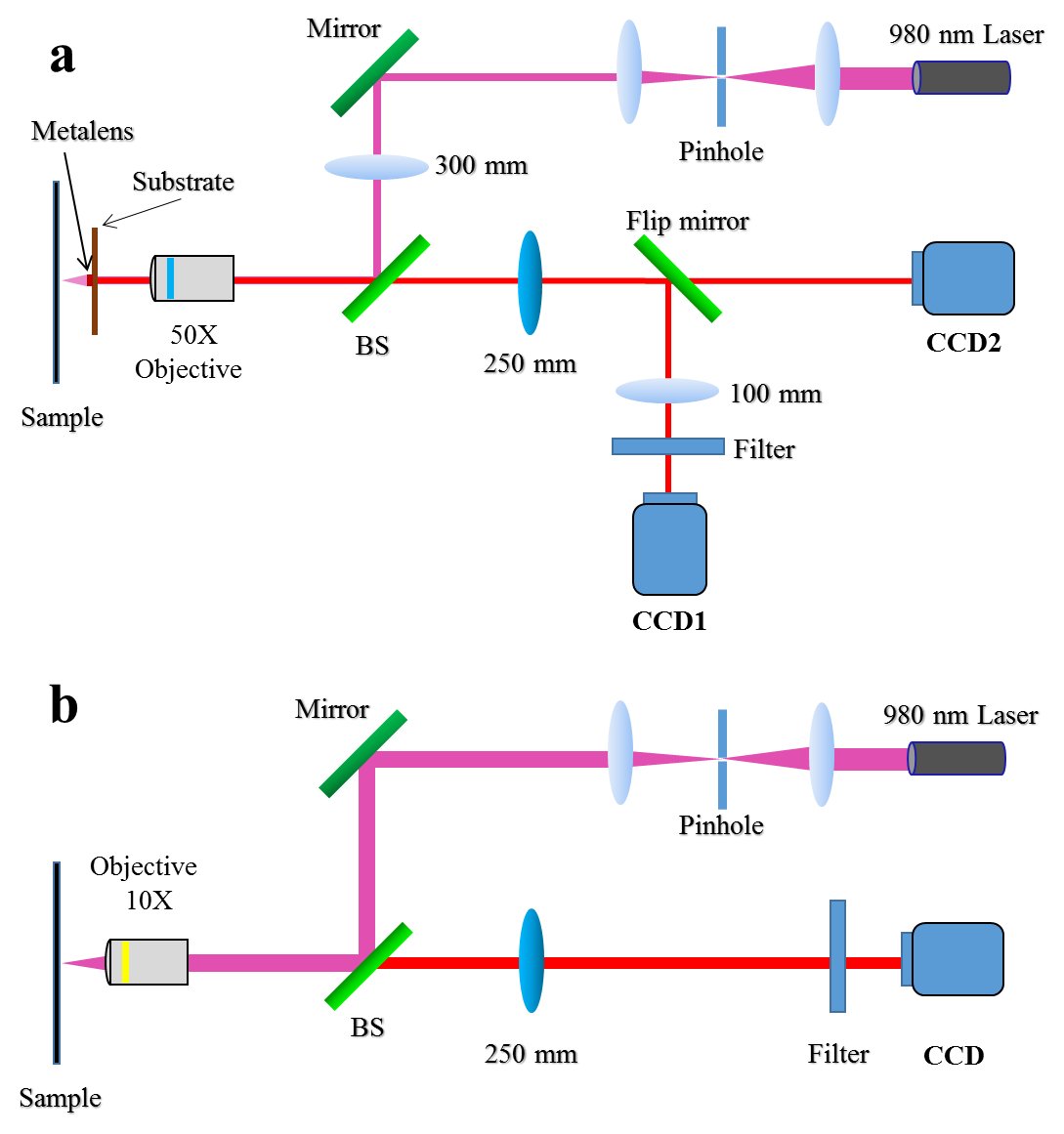


**Supplementary Figure 9. The optical setups for upconversion imaging. a** Up conversion imaging optical setup. It is a confocal scanning microscope consist of two imaging sub-modules. A flip mirror is used to switch between them. **b** Up conversion imaging light path for 10X objective lens is a variant of metalens version. By removing 300 mm lens, a classical confocal microscope is formed. 980 nm laser over filled the 10X achromatic objective lens. All the optical components are achromatic in the operation wavelength range.

In the main text, we have determined that the two-photon imaging with TiO_2_ metalens can capture the structural information of a microplate embedded in scattering PS spheres. The image quality is as good as a commercial achromatic objective lens. Here we show a series of experiments to further confirm this information. Supplementary Figure 10 and Supplementary Figure 11 show the optical images of two triangular microplates. By comparing the images recorded with a metalens and a commercial objective lens, we can conclude that the metalens can produce the same quality as the commercial objective lens. This characteristic is very essential for practical applications. The high-quality imaging capability, associated with the ultracompact size, is able to revolutionize many related areas, e.g. endoscope and untethered mini- or micro-robots.


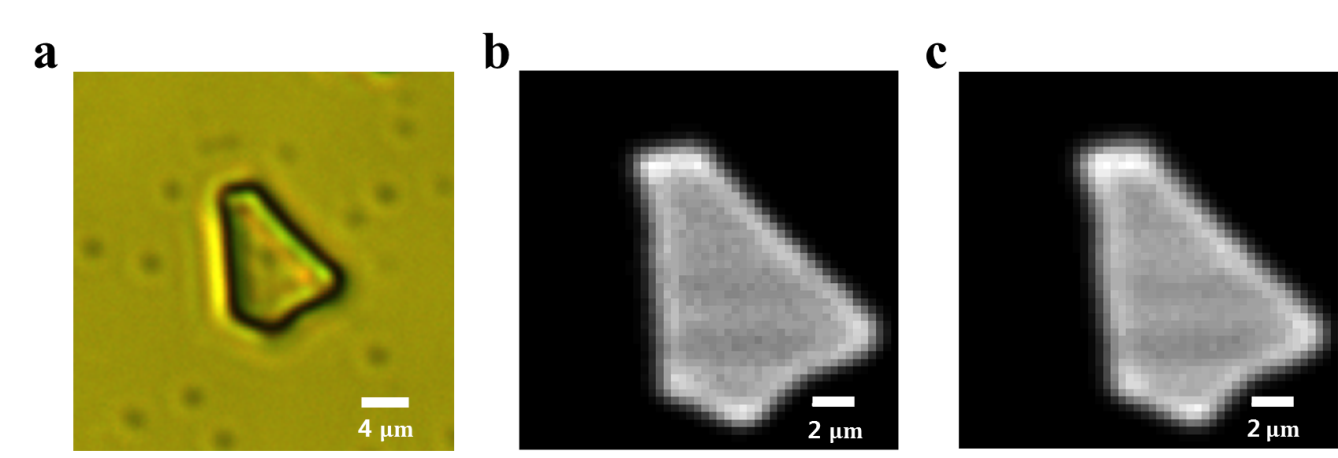


**Supplementary Figure 10.** **The upconversion fluorescent imaging of one triangle microplate. a** The microscope image of NCs based microplate under the white light illumination. **b** The two-photon emission image recorded by the achromatic metalens with NA = 0.24. **c** The two-photon emission image recorded by a commercial achromatic objective lens with NA = 0.26 (MY10X-823, Mitutoyo).


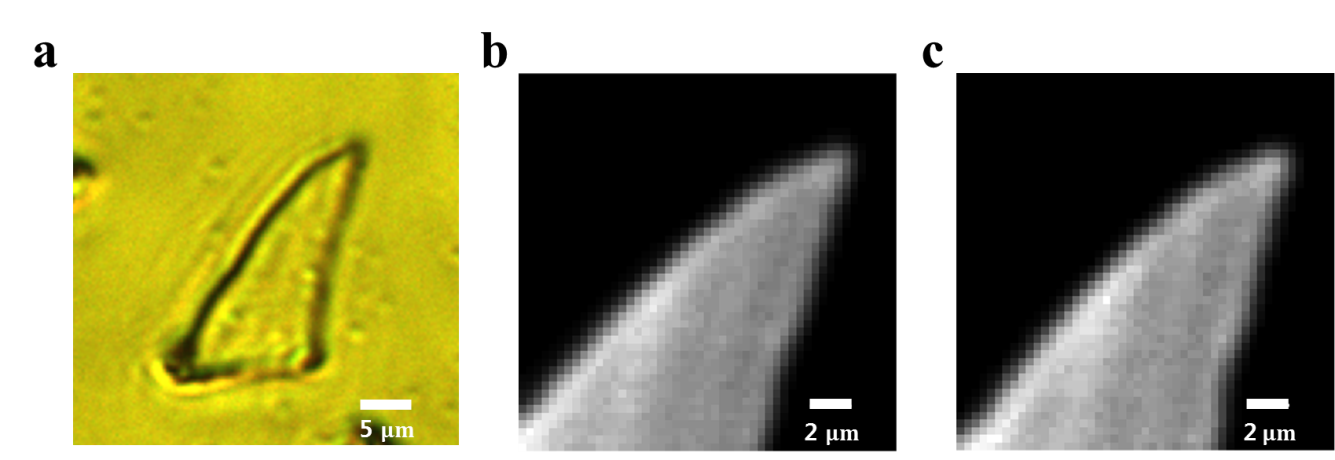


**Supplementary Figure 11.** **The upconversion fluorescent imaging of another triangle microplate. a** The microscope image of NCs based microplate under the white light illumination. **b** The two-photon emission image recorded by the achromatic metalens with NA = 0.24. **c** The two-photon emission image recorded by a commercial achromatic objective lens with NA = 0.26 (MY10X-823, Mitutoyo).

**Supplementary Note 8: The working wavelength range**

As depicted in Supplementary Figure 1, TiO_2_ has high refractive index and low absorption in the entire visible spectrum the near IR region. In our research, we only demonstrate the one to fill the low efficiency gap in previous achromatic metalens. From the point view of optical properties, TiO_2_ has the potential to construct high performance achromatic metalens with a wavelength down to ~400 nm. To demonstrate such a potential, we have also designed a metalens for the visible spectrum. With the reduction of operation wavelength, the thickness of TiO_2_ can be reduced to 1200 nm. Based on the same process as the above, we have designed an achromatic metalens with a diameter of 25 µm and NA of 0.2. Supplementary Figure 12a shows a quarter of the metalens. The TiO_2_ nanopillars have six types of geometries that have 4-fold or rotational symmetry. The corresponding numerically calculated results are depicted in Supplementary Figure 12b and Supplementary Figure 12c. By maintaining the focal length at 60 µm, the averaged efficiency can be preserved at a higher value of 88.3%.

It is important to note that the spectral range of the achromatic metalens cannot be infinitely extended. In principle, there is a trade-off between the NA, device size, and spectral range for a fixed range of group delay. For all the designs in the manuscript, the group delay is optimized with the high transmittance. As a result, it can maintain the high performance either in the visible spectrum or in the near IR biological imaging window. There is indeed a possibility of fully covering the visible and near IR spectral range. This can be realized by increasing the range of group delay. Similar to the previous works, we can extend the group delay to a much larger range without considering the transmittance. In this case, we have also designed a metalens for the spectrum from 400 nm to 1500 nm. However, the overall efficiency is strongly spoiled to below 30%. Therefore, we are focusing on the metalens for a particular spectrum in experiments.


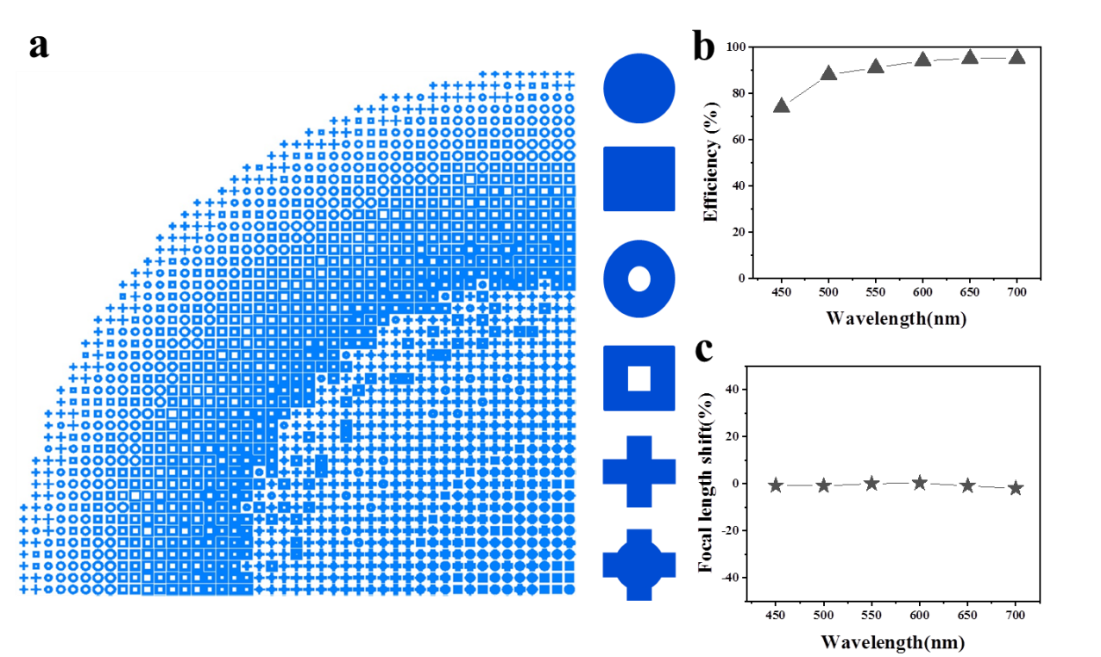


**Supplementary Figure 12. The design of TiO_2_ achromatic metalens for the visible spectrum. a** The layout of a quarter of the metalens. Six types of nanopillars are employed. **b** and **c** are the numerically calculated focal length and the efficiency. Here the diameter and NA of the metalens are 25 µm and 0.2, respectively.
